# Atomic force microscopy-based indentation of cells: modelling the effect of a pericellular coat

**DOI:** 10.1098/rsif.2022.0857

**Published:** 2023-02-22

**Authors:** Ivan Argatov, Xiaoqing Jin, Gennady Mishuris

**Affiliations:** ^1^ College of Aerospace Engineering, Chongqing University, Chongqing, 400030, People’s Republic of China; ^2^ Institut für Mechanik, Technische Universität Berlin, 10623 Berlin, Germany; ^3^ Department of Mathematics, Aberystwyth University, Ceredigion SY23 3BZ, Wales, UK

**Keywords:** atomic force microscopy indentation, living cell, pericellular brush, mathematical modelling

## Abstract

A simple analytical model is built up to account for the interface deformation effect in a spherical atomic force microscopy (AFM)-based quasi-static indentation of a living cell covered with a pericellular brush. The compression behaviour of the pericellular coat is described using the Alexander–de Gennes model that allows for nonlinear deformation. An approximate second-order relation between contact force and indenter displacement is obtained in implicit form, using the Hertzian solution as a first-order approximation. A method of fitting the indentation brush/cell model to experimental data is suggested based on the non-dimensionalized version of the displacement–force relation in the parametric form and illustrated with a specific example of AFM raw data taken from the literature.

## 1. Introduction

It is well known that qualitative and quantitative changes of mechanobiological markers, such as extracellular matrix stiffness [1], cell adhesion [2] and cell Young’s modulus [3] are closely related to the physiological state of cells and can be associated with diverse pathologies [4,5].

Atomic force microscopy (AFM) [6] represents a convenient tool for mechanical testing at the single-cell level [7,8]. To increase its sensitivity in detection of a pericellular coat on eukaryotic cells, it was suggested to use a spherical probe instead of typical sharp pyramidal indenters [9]. (A detailed description of the AFM indentation technique for studying cell mechanics and pericellular coat is available elsewhere [10].)

A key point in effective application of AFM in mechanobiology is an appropriate mathematical model for interpreting AFM raw data (deflection of the AFM cantilever, *d*, with respect to relative displacement of the AFM piezo-scanner, *Z*, which describes the vertical position of the AFM cantilever base). With a known bending stiffness of the AFM cantilever, *k*_c_, the contact force, *F*, exerted by an AFM probe (indenter) on the surface of a tested cell is usually evaluated by means of the linear relation *F* = *k*_c_*d*. A much more difficult challenge is to relate the *absolute* (i.e. with respect to the laboratory frame) scanner displacement *Z* to the *absolute* indenter (probe) displacement, *δ*, because the latter quantity reflects the deformation response of the brush/cell system. At the same time, the mechanobiological markers can be revealed via the analysis of the force–displacement curve (*F* versus *δ*).

**Figure 1.**
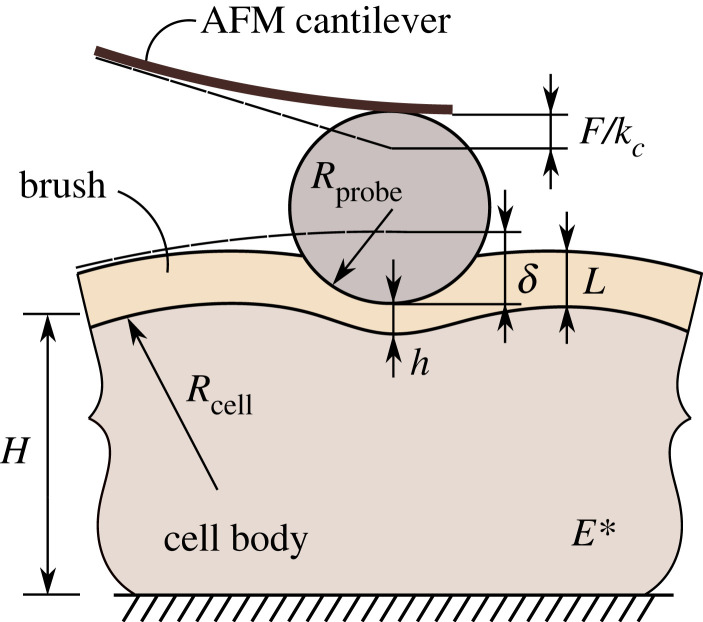
Schematic of the AFM-based indentation of a cell with a pericellular coat (brush). *Cell body:* *R*_cell_ is the curvature radius of the cell’s surface, *H* is the characteristic cell thickness, E* is the reduced elastic modulus. *Brush layer:* *L* is the brush thickness. *Atomic force microscopy, (AFM) model:* *k*_c_ is the cantilever bending stiffness, *R*_probe_ is the radius of the AFM indenter. *Contact variables:* *F* is the contact force, *δ* is the indenter displacement, and *h* is the separation distance between the AFM tip and the surface of the cell.

The choice of a correct contact model depends on a number of factors such as a constitutive equation for the cell body, the shape of indenter, the cell geometry and the interface conditions among others. In particular, in the case of a spherical AFM probe (figure 1), the classical Hertz model is often used with no regard for the assumptions which the model is based on. We recall [11,12] that the Hertzian force–displacement relation takes the form

1.1 $$F=\frac{4}{3}E^{*}\sqrt{R}\delta^{3/2},$$

where *R* is the effective curvature radius (which, besides the probe radius, *R*_probe_, also accounts for the local curvature radius, *R*_cell_, of the cell surface at the point of indentation), and E* is the so-called effective elastic modulus (which, besides the cell Young’s modulus, *E*, and Poisson’s ratio, *ν*, may also account for the elasticity of the probe). In the analysis presented below, the indenter elasticity is neglected, and therefore, E* becomes the reduced elastic modulus, which is also called the indentation modulus [13].

It is important to highlight that equation (1.1) adopts the linearly elastic half-space approximation for evaluating the contact deformations of a cell in the region near the contact zone [11]. Moreover, when equation (1.1) is applied to the analysis of the AFM-based testing of living cells, it is tentatively assumed that cells have bare surfaces without being covered by any membrane protrusions or corrugations [14,15]. The latter simplifying assumption has been criticized for not being realistic enough for many types of living cells that are coated with pericellular brushes [14] and the so-called brush model has been introduced [16], which complements equation (1.1) by the model of entropic brush in the exponential form [17,18] for the relation between the contact force *F* and the separation distance, *h*, of the indenter tip from the brush grafting surface.

In the present study, we make use of a general nonlinear constitutive equation for describing the compression deformation of a pericellular coat. The kinematic contact condition takes into account the simultaneous contact deformation of the brush layer and the cell body. The resulting problem for the contact pressure is formulated as a nonlinear integral equation, and an approximate analytical solution for the displacement–force relation is obtained in the form of a system of coupled nonlinear equations. A parametric analysis of the developed model is performed, and an example of fitting the model to a set of experimental data taken from the literature is outlined.

Generally speaking, the force–displacement relation in the indentation of a brush/cell system can be represented in the parametric form as

1.2 $$F=F_{1}(a;E^{*},p_{1})$$

and

1.3 $$\delta =F_{2}(a;E^{*},p_{1}),$$

where *a* is the contact radius, E* and *p*_1_ are characteristic (effective) stiffness parameters of the cell body and the brush layer, respectively, both of which have the physical dimension of pressure, and $F_{1}$, $F_{2}$ are two functions given in explicit form. It should be emphasized that our model aims at evaluating both the effective reduced elastic modulus E* of the cell body and the stiffness parameter *p*_1_ of the pericellular coat. It should be noted that the latter characteristic is also found to be useful in mechanobiological research for discriminating between normal and abnormal cells [19]. Moreover, it should be underlined that though the brush parameter *p*_1_ has the same dimension as the cell reduced elastic modulus E*, the mechanical interpretation of these stiffness characteristics is different (see [20]).

Since the contact radius *a* cannot be directly measured in the AFM-based indentation, the use of equations (1.2) and (1.3) for analysis of experimental data is not straightforward. In many cases, it is convenient to regard the contact force *F* as a primary variable, and equation (1.2) can be resolved for *a* to obtain

1.4 $$a=F_{1}^{-1}(F;E^{*},p_{1}),$$

where $F_{1}^{-1}$ is the inverse function of the function $F_{1}$. Then, the substitution of the above expression into equation (1.3) yields the indenter displacement *δ* as an explicit function of *F*. The main difficulty in this approach is the lack of analytical representation for the inverse function $F_{1}^{-1}$ in equation (1.4), and therefore, equation (1.2) should be solved numerically. However, this can be accomplished in an efficient manner using standard computational software.

In the indentation brush/cell model developed below, the problem with the force–displacement relation is even more difficult, because the three contact variables *F*, *δ* and *a* are connected by two equations in the implicit form

1.5 $$G_{1}(F,a;E^{*},p_{1})=0$$

and

1.6 $$G_{2}(F,a;E^{*},p_{1})=\delta ,$$

where $G_{1}$, $G_{2}$ are two functions given in explicit form. Nevertheless, by numerically solving equation (1.5) for *a* and substituting the obtained result into equation (1.6), we again obtain the explicit relation between the contact force *F* and the indenter displacement *δ*.

The rest of the paper is organized as follows. In §2, we give a technical introduction to the indentation brush/cell model and derive the force–displacement relation in implicit form. In §3, we represent the model in the non-dimensionalized form and outline the method of fitting the model to experimental data. Finally, in §4, we discuss the constructed model, some of its generalizations, and formulate our conclusions. Electronic supplementary material contains parametric analyses of both the indentation brush model and the indentation brush/cell model.

## 2. Theory

### 2.1. Nonlinear compliance model for a pericellular brush

As it was mentioned in the introduction, the majority of living cells are covered with protrusions and corrugations of diverse shapes and sizes [21]. This means that modelling the mechanical deformation of a pericellular brush from first principles is very challenging and not always feasible due to lack of specific data. On the other hand, in the literature there is a number of simple models applicable to monodisperse polymer brushes [22,23]. In particular, the Alexander–de Gennes (AdG) theory [24,25] predicts that the quasi-static compression of a grafted polymer brush of uniform thickness, *L*, in contact with a rigid planar surface is governed by the equation

2.1 $$p=p_{1}f\left(\frac{D}{L}\right),\;0<D\leq L.$$

Here, *p* is the compression pressure, *D* is the distance between the two rigid surfaces (one of which is a brush grafting surface), *p*_1_ ∼ *k*_B_*T*/*s*^3^ with *s* being the average distance between grafting points, and in the AdG theory, we have *f*(*λ*) = *f*_AdG_(*λ*), where

2.2 $$f_{\mathrm{AdG}}(\lambda )=\lambda^{-9/4}-\lambda^{3/4},$$

and *λ* = *D*/*L* is the stretch ratio.

We note that the flexibility of the phenomenological model (2.1) can be increased by adopting the two-parameter approximation $f\;(\lambda )=\lambda^{-\nu_{1}}-\lambda^{\nu_{2}}$, where *ν*_1_ and *ν*_2_ are positive fitting constants. A physical motivation for the choice of the AdG model for describing deformations of a pericellular coat is given in [14]. It should be emphasized that the AdG model has been represented in the generalized form of equation (2.1), and the specific law (2.2) for the constitutive function *f*(*λ*) is used only in considering a specific example of experimental data.

It is also pertinent to note here that the variable *D* has a geometrical meaning of the brush thickness in the loaded state, and therefore, in a progressive compression *D* decreases.

Observe that the prefactor *p*_1_ has a physical dimension of pressure, which is the same as that of Young’s modulus of elasticity. That is why, when equation (2.1) is applied to polydisperse brushes, *p*_1_ can be interpreted as a characteristic modulus, while *L* is treated as an effective brush thickness.

In what follows, we need the inverse constructive relation

2.3 $$D=Lf^{-1}\left(\frac{\;p}{p_{1}}\right),\;0\leq p<\infty ,$$

where *f*^−1^ is the inverse function to *f*. According to the unloaded equilibrium condition, we have *f*(1) = 0, from where it follows that *f*^−1^(0) = 1. We note that in the case of the AdG model (2.2), the evaluation of *f*^−1^ can be reduced to solving a quartic algebraic equation.

As it was noted [17], the constitutive function (2.2) is roughly exponential in the range from 0.2 to 0.9 (see electronic supplementary material, figure S9), i.e.

2.4 $$f_{\mathrm{AdG}}(\lambda )\approx 100\exp(-2\pi \lambda ).$$

The exponential approximation (2.4) is widely used for fitting experimental data due to its simplicity [18,26]. However, it cannot be applied for modelling the initial contact between the brush layer and an indenter.

### 2.2. Hertz-type contact with a brush-like interface

To simplify the mathematical analysis, we consider the axisymmetric contact configuration (referred to cylindrical coordinates *r*, φ and *z*) and employ the following Boussinesq’s solution to approximate (normal) surface elastic displacements of a cell:

2.5 $$u_{z}(r)=\frac{1}{\pi E^{*}}\int_{0}^{2\pi }\;d\phi \int_{0}^{a}\frac{\;p(\rho )\rho \;d\rho }{\sqrt{r^{2}+\rho^{2}-2r\rho \cos\phi }}.$$

Here, E* = *E*/(1 − *ν*^2^) is the so-called reduced elastic modulus (with *E* and *ν* being the cell Young’s modulus and Poisson’s ratio, respectively), *p*(*r*) is the density of normal surface loads, *ϕ* is the integration variable (the left-hand side of equation (2.5) does not depend on the angular coordinate φ of the point of observation), *a* is the radius of a loaded region and *ρ* is the integration variable. For our purposes, we make use of formula (2.5) only for approximating the surface displacements inside the circular area 0 ≤ *r* ≤ *a*.

It should be made clear that the cell thickness parameter *H* does not enter any formula of the analysis below. This means that the cell thickness effect or the so-called bottom effect has been neglected. In other words, the contact deformations of the cell body are evaluated by treating it as an elastic half-space and using Boussinesq’s solution (2.5). However, following the asymptotic modelling approach [27,28], we can account for the main contribution of the bottom effect in the case of a relatively thick cell body (i.e. roughly speaking when *R* < *H*). This generalization will be published elsewhere.

We note also that the bottom-effect models, which are specifically adopted for AFM indentation experiments, have been recently developed in the literature [29,30]. A more general asymptotic analysis of the thickness/substrate effect (for indenter of arbitrary axisymmetric convex shape and transversely isotropic material properties of the layer/substrate system) was presented in [28]. The main difference of our present approach to the brush effect compared with those mentioned above is the specific deformation response of the brush layer, which acts according to a nonlinear Winkler foundation model.

**Figure 2.**
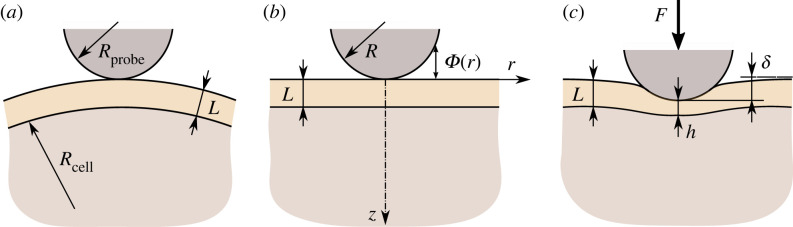
Schematic of the unilateral contact between a spherical probe of radius *R*_probe_ and an elastic cell of curvature radius *R*_cell_ covered by a pericellular brush of thickness *L*: (*a*) unloaded state with a single point contact; (*b*) equivalent model for an elastic half-space covered by a nonlinear Winkler-type coating and indented by a spherical indenter of effective radius *R* that defines the initial gap Φ(r) (see equation (2.6)); (*c*) loaded state: the brush thickness outside the contact zone remains the same as in the unloaded state.

Let Φ(r) denote the initial gap between the brush surface and the surface of the AFM probe in the unloaded state when the two surfaces are brought into a single point contact (figure 2*a*). For a spherical probe of radius *R*_probe_, the following paraboloidal approximation is usually employed:

2.6 $$\Phi (r)=\frac{r^{2}}{2R},$$

where *R* is the one-half of the harmonic mean of *R*_probe_ and the curvature radius of the cell surface, *R*_cell_, that is *R* = *R*_probe_*R*_cell_/(*R*_probe_ + *R*_cell_). It should be emphasized that the utilization of Boussinesq’s solution (2.5) in the framework of the Hertzian contact mechanics does not distinguish between the two contact geometries shown in figure 2*a*,*b*.

In the loaded state (figure 2*c*), the indenter receives some normal (vertical) displacement, *δ*, under the action of an external force, *F*. According to the equilibrium equation, we have

2.7 $$F=2\pi \int_{0}^{a}p(r)r\;dr.$$

Now, let *D*(*r*) denote the variable thickness of a pericellular brush, which is squeezed between both brush/probe and brush/cell interfaces. Since contact between the AFM probe and the brush surface is assumed to be unilateral and non-adhesive, the radius of contact *a* is determined by the condition

2.8 p(a)=0,

which, in view of equation (2.3), implies that

2.9 D(a)=L.

The kinematic contact condition,

2.10 $$D(r)=L+u_{z}(r)-\delta +\Phi (r),$$

states that the thickness of the compressed brush is determined by a balance of displacements due to the indenter-induced vertical-downward displacements δ−Φ(r) at the brush interface and the cell displacements *u*_*z*_(*r*) at the brush bottom surface.

**Figure 3.**
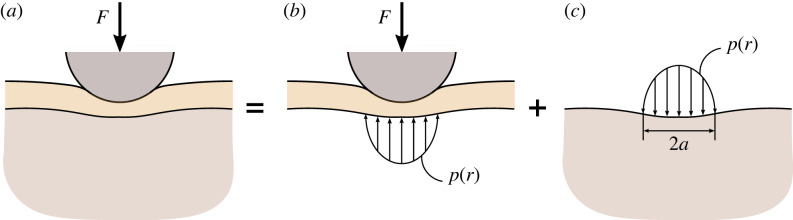
Schematic of the brush/cell equilibrium in the loaded state: (*a*) the external contact load *F* is distributed by means of the indenter on the surface of the brush layer; (*b*) the brush layer transfers the distributed load, which is characterized by the pressure density *p*(*r*), to the surface of the cell; (*c*) the only surface load that acts on the cell is the contact pressure *p*(*r*), distributed over a circular area of *a priori* unknown radius *a*.

Figure 3 shows a schematic of the equilibrium in the brush/cell system in the loaded state. Since the brush layer does not exhibit any shear resistance, the contact pressure *p*(*r*) will be directly transmitted from the indenter/brush interface to the brush/cell interface. This explains the use of the same contact radius in equations (2.9) and (2.5) for the radius of the indenter/brush contact region and the radius of the loaded region at the brush/cell interface, respectively. It is to emphasize that in figure 3, we have employed the general property of brush-like models that the brush layer does not transfer any shear load, and as such, the contact pressure produced by the indenter on the brush top surface is transferred (without changes) to the cell top surface.

Further, according to the inverse constitutive equation (2.3), we have

2.11 $$D(r)=Lf^{-1}\left(\frac{\;p(r)}{p_{1}}\right),$$

thereby relating the variable thickness of the deformed brush layer *D*(*r*) to the contact pressure *p*(*r*).

Thus, from equations (2.5), (2.10) and (2.11), it follows that

2.12 $$L\left\{1-f^{-1}\left(\frac{\;p(r)}{p_{1}}\right)\right\}+\frac{1}{\pi E^{*}}\left(Bp\right)(r)=\delta -\Phi (r),$$

where (Bp)(r) denotes the integral operator on the right-hand side of equation (2.5), i.e.

2.13 $$\left(Bp\right)(r)=4\int_{0}^{a}K\left(\frac{2\sqrt{\rho r}}{\rho +r}\right)\frac{\;p(\rho )\rho }{\left(r+\rho \right)}\;d\rho ,$$

and **K**(*x*) is the complete elliptic integral of the first kind.

We note that equation (2.12), which represents the governing integral equation of the indentation problem, can be reduced to a nonlinear integral equation of the Hammerstein type. In the next sections, we construct an approximate solution to equation (2.12) satisfying the boundary condition (2.8), which allows us to derive an analytical approximation for the relation between the contact force *F* and the indenter displacement *δ*.

### 2.3. Analysis of the limit cases

Observe that the governing integral equation (2.12) describes the simultaneous deformation of the brush layer and the cell body. It is known [16] that the pericellular coat is often much softer than the cell body, meaning that the indenter displacement effect might not be transmitted to the cell surface under a relatively small level of indentation. In such a case, the second term on the left-hand side of equation (2.12) is negligible, and thus, the integral equation reduces to the algebraic equation $L\{1-f^{-1}(p(r)/p_{1})\}=\delta -\Phi (r)$, which describes the deformation of solely the brush layer. This is in complete agreement with the above hypothesis for the experimentally established relative brush/cell stiffness. However, as the level of indentation increases, the effect of the second term on the left-hand side of equation (2.12) becomes increasingly important, and meanwhile there is an intermediate indentation range at which the two terms on the left-hand side of equation (2.12) are of the same order.

In the absence of a brush layer, as *L* tends to zero, equation (2.12) reduces to the governing integral equation of the Hertz theory implying the relation between contact force and indenter displacement in the form of equation (1.1), which is complemented by the following relation between contact radius and indenter displacement:

2.14 $$a=\sqrt{R}\delta^{1/2}.$$

The inverse relation to equation (1.1) takes the form

2.15 $$\delta ={\left(\frac{3}{4E^{*}\sqrt{R}}\right)}^{2/3}F^{2/3},$$

and represents the displacement–force curve.

By differentiating both sides of equation (2.15) with respect to the contact force *F*, we evaluate the incremental indentation compliance

2.16 $$\frac{d\delta }{dF}=\frac{2}{3}{\left(\frac{3}{4E^{*}\sqrt{R}}\right)}^{2/3}F^{-1/3}.$$

We note that usually the incremental indentation stiffness d*F*/d*δ* is used in indentation testing (e.g. [13,31]). However, a series connection of brush and cell, which are subjected to normal indentation, warrants the use of namely the indentation compliance.

On the other hand, in the limit as E* tends to infinity, when the brush/cell system reduces to a brush layer grafted on a rigid substrate, the integral term in equation (2.12) disappears and the latter simplifies to the equation

2.17 $$p(r)=p_{1}f\left(\frac{\delta -\Phi (r)}{L}\right),$$

which, in turn, in view of (2.8), implies the equation Φ(a)=δ for evaluating the contact radius *a*.

In the case of a paraboloidal indenter with the shape function (2.6), we have

2.18 $$a=\sqrt{2R}\delta^{1/2}.$$

By integrating the contact pressure density (2.17) over the circular contact region of radius (2.18) (see e.g. [20]), we can arrive at the following force–displacement relation [23,32]:

2.19 $$F=2\pi p_{1}RL\int_{(L-\delta )/L}^{1}f\;(\lambda )\;d\lambda .$$

Moreover, based on the exponential approximation (2.4), the following Derjaguin’s approximation can be deduced [18]:

2.20 $$F_{\mathrm{AdG}}\approx 100p_{1}RL\exp\left(-2\pi \frac{h}{L}\right).$$

Here, *h* is the thickness of the compressed brush beneath the indenter tip, i.e.

2.21 h=L−δ.

It can be shown (see electronic supplementary material) that the exponential approximation (2.20) is fairly accurate in the range from 0.2 to 0.7 for the relative contact brush thickness *h*/*L*.

Further, by differentiating both sides of equation (2.19) with respect to the indenter displacement, we evaluate the brush incremental indentation stiffness as d*F*/d*δ* = 2*πp*_1_*Rf*(1 − *δ*/*L*), so that the incremental indentation compliance can be expressed in the form

2.22 $$\frac{d\delta }{dF}={\left(2\pi p_{1}Rf\left(1-\frac{\delta (F)}{L}\right)\right)}^{-1},$$

where *δ*(*F*) denotes the solution of equation (2.19). We note that in the case of the exponential approximation (2.20), in view of (2.21), we have *δ*(*F*) = *L* + (*L*/2*π*)ln(*F*/100 *p*_1_*RL*).

To compare the two limit models, we adopt the following characteristic values for the model parameters [14]: *E* = 2.1 kPa, *ν* = 0.5, *L* = 2.36 μm, and *R* = 5 μm. Also, based on the average value *N* = 290 brush molecules per μm^2^ for the grafting density, we have estimated the AdG prefactor as *p*_1_ = 10.6 Pa.

**Figure 4.**
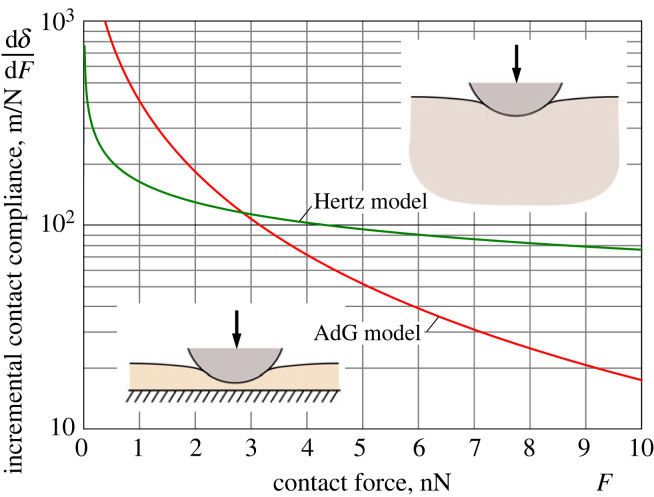
Incremental indentation compliances (2.16) and (2.22) for the Hertz model (green solid line) and the Alexander–de Gennes (AdG) indentation model (red solid line), respectively, as functions of contact force. The model parameters are specified as follows: E* = 2.8 kPa, *p*_1_ = 10.6 Pa, *L* = 2.36 μm and *R* = 5 μm.

Figure 4 shows the behaviour of the indentation (contact) compliances (2.16) and (2.22). Observe that, as it should be expected, both incremental indentation compliances decrease by virtue of the fact that the contact area (which is produced by paraboloidal indenter) increases with the level of indentation. It is of paramount importance that the two curves in figure 4 cross each other at a single point $F_{*}$ approximately 3 nN. This means that in the initial period of loading (for $F<F_{*}$), the brush layer exhibits a relatively large compliance compared with that of the elastic cell body. Hence, the displacement of the AFM indenter pressed into an elastic body covered with a brush (that is, in the case when the two systems are connected in series) would be primarily accommodated via the brush compression deformation. In the advanced stage of loading (for $F>F_{*}$), the Hertzian compliance is larger by approximately an order of magnitude than the indentation compliance of the brush layer, so that the Hertzian contribution to the indenter displacement now becomes dominant.

**Figure 5.**
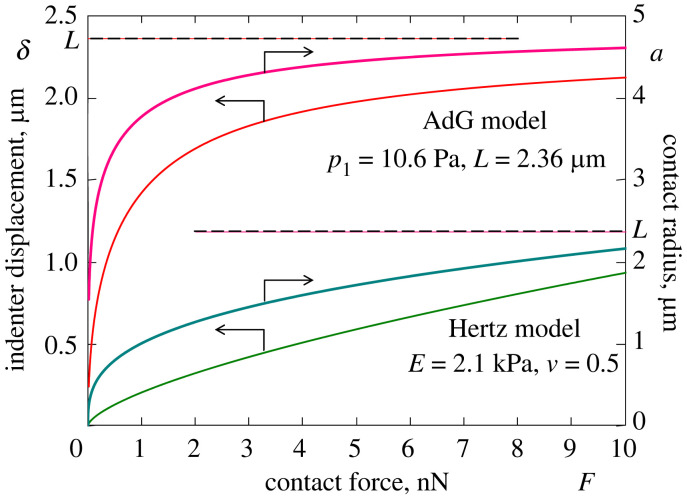
Indenter displacement (left scale) and contact radius (right scale) as functions of contact force for the Hertz model (two lower solid lines of greenish colour; see equations (2.15) and (2.14), respectively) and the Alexander–de Gennes (AdG) model (two upper solid lines of reddish colour; see equations (2.19) and (2.18), respectively). The model parameters are the same as in figure 4. The two dashed lines denote the assumed value for the brush layer thickness.

In indentation testing, neither E* nor *p*_1_ is known *a priori*, and therefore, it is not possible to draw the curves in figure 4 in the physically dimensional variables given only the set of AFM indentation data and without its post-processing using an appropriate mathematical model. The corresponding *δ*–*F* and *a*–*F* curves are presented in figure 5. The most important lesson learned from this figure is that the contact radii for the two models, which correspond to the same value of the contact force, are essentially different. This means that the Hertz model and the AdG model, which are regarded as the sub-models of the indentation brush/cell model, do not work independently.

Yet another limit situation occurs when the brush layer is much stiffer than the cell body. From a mathematical point of view, we consider the governing integral equation as *p*_1_ tends to infinity, so that equation (2.12) reduces to the governing equation of the Hertz theory.

**Remark.**

In the mechanobiological literature [16,21], the concept of cell modulus of elasticity was introduced in connection to AFM indentation testing. When the Hertz theory-based formula $dF/d\delta =2E^{*}\sqrt{R\delta }$ is used for the interpretation of the incremental indentation stiffness *S* = d*F*/d*δ*, evaluated with a spherical indenter, the quantity $E^{*}=S/(2\sqrt{R\delta })$ should be relatively independent of indentation depth in order to serve as an effective material characteristic of the cell body. Thus, namely, the plateau of the relative incremental stiffness curves (see electronic supplementary material, figure S13) is decisive for determining E* rather than their initial part influenced by the pericellular coat. However, even though theoretically the impact of the brush layer tends to zero for larger indentation depth, practically it is not always possible to effectively minimize the brush effect. That is why the use of the brush/cell model allows to increase the reliability of the determination of the effective cell modulus.

### 2.4. Displacement–force relation in implicit form

In solving the indentation problem (2.9), (2.12), we take advantage of the experimentally supported assumption [14] that the brush is softer then the cell body. This means that the first term on the left-hand side of equation (2.12) can be neglected as a zero-order approximation, and we arrive at the Hertz solution

2.23 $$p(r)=\frac{3F}{2\pi a^{2}}\sqrt{1-\frac{r^{2}}{a^{2}}}.$$

Now, the substitution of (2.23) into equation (2.12) leads to an approximate relation, which can be used to produce a first-order approximation. In this way, by reinforcing thus derived approximate relation at the centre of the contact area (*r* = 0) and the contact contour (*r* = *a*), we derive the approximate equations

2.24 $$L\left\{1-f^{-1}\left(\frac{3F}{2\pi a^{2}p_{1}}\right)\right\}+\frac{3F}{8E^{*}a}=\frac{a^{2}}{2R}$$

and

2.25 $$\frac{3F}{8E^{*}a}+\frac{a^{2}}{2R}=\delta .$$

The coupled system of equations (2.24) and (2.25) constitutes the displacement–force relation in implicit form. Namely, for a given value of the contact force *F*, equation (2.24) determines the corresponding value of the contact radius *a*, and then substituting both *F* and *a* into equation (2.25) yields the indenter displacement *δ*.

According to equations (2.11) and (2.23), the brush layer thickness at the centre of the contact area, *h* = *D*(0), will be given by

2.26 $$h=Lf^{-1}\left(\frac{3F}{2\pi a^{2}p_{1}}\right),$$

from where, in view of equations (2.24) and (2.25), it follows that

2.27 $$h=L+\delta -\frac{a^{2}}{R}.$$

It is also of interest to evaluate the cell contact displacement $u_{z}^{0}=u_{z}(0)$, which according to equation (2.12) can be approximated as

2.28 $$u_{z}^{0}=\frac{3F}{4E^{*}a}.$$

It should be emphasized that the Hertz model is recovered from equations (2.24) and (2.25) in the limit as *L* tends to zero or *p*_1_ tends to infinity. Moreover, it can be easily verified that equation (2.25) is in complete agreement with the Hertz model. On the other hand, it can be shown that equation (2.27) agrees with Derjaguin’s approximation for a brush layer, which implies that *h* = *L* − *δ* and *a*^2^ = 2*Rδ*. Indeed, the substitution of the latter expressions into equation (2.27) leads to an identity.

## 3. Results

### 3.1. Indentation model in the non-dimensionalized form

Let us introduce the dimensionless variables

3.1 $$\bar{a}=\frac{a}{R},\;\bar{\delta }=\frac{\delta }{R}\;\mathrm{and}\;\;\bar{F}=\frac{3F}{8E^{*}R^{2}},$$

and the dimensionless parameters

3.2 $$\bar{L}=\frac{L}{R}\;\mathrm{and}\;\chi^{*}=\frac{4E^{*}}{\pi p_{1}}.$$

Then, equations (2.24) and (2.25), respectively, can be represented in the form

3.3 $$\chi^{*}\frac{\bar{F}}{{\bar{a}}^{2}}=f\left(1-\frac{{\bar{a}}^{3}-2\bar{F}}{2\bar{L}\bar{a}}\right)$$

and

3.4 $$\frac{\bar{F}}{\bar{a}}+\frac{{\bar{a}}^{2}}{2}=\bar{\delta }.$$

We note that by transforming equation (2.24) to equation (3.3), we get rid of the inverse function *f*^−1^ to simplify its numerical solution. Observe that equations (3.3) and (3.4) contain two dimensionless parameters, namely, χ* and $\bar{L}$ that govern the portrait of the force–displacement relation.

### 3.2. Fitting the model to experimental data

**Figure 6.**
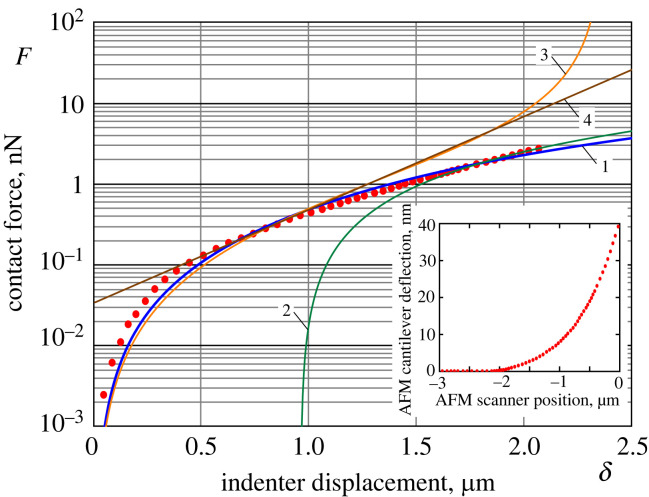
An example of processing raw data [10] (see the insert), deflection of the AFM cantilever versus vertical position of the AFM scanner (red dotted line). Solid lines 1, 2, 3 and 4, respectively, denote the present model (blue line, 1), the Hertz model (green line, 2), the AdG indentation model (2.2), (2.19) (orange line, 3) and the exponential model (2.20) (brown line, 4). The dotted red line of the main graph represents the processed loading part of the insert graph.

We consider one example of raw data (see the insert in figure 6) from AFM-based indentation of an eukaryotic cell with a 5 μm silica particle as the AFM probe, which was published in [10]. The cantilever spring constant is taken to be *k*_c_ = 0.069 N m^−1^ [33]. We note that the contact force *F* on the main ordinate axis is evaluated from the AFM cantilever deflection *d* depicted on the ordinate axis of the insert graph by the formula *F* = *k*_c_*d*. The effective radius *R* is assumed to be coincident with the probe radius *R*_probe_, since the effect of the cell curvature radius *R*_cell_ is relatively small.

The numerical solution of the system of equations (3.3) and (3.4) was carried out within the Mathcad software environment. In what follows, the numerical solution of equation (3.3) will be denoted as

3.5 $$\bar{F}=F(\bar{a},\chi^{*}),$$

so that equation (3.4), in view of equation (3.5), yields

3.6 $$\bar{\delta }=\frac{1}{\bar{a}}F(\bar{a},\chi^{*})+\frac{{\bar{a}}^{2}}{2}.$$

According to equations (3.1) and (3.2), formulae (3.5) and (3.6) contain the two-dimensionless parameters χ* and $\bar{L}$. Yet, another dimensional parameter, *F*_1_, is introduced to provide the dimensional scaling for the force data. Thus, the force–displacement relation in the parametric form can be represented as follows:

3.7 $$F=F_{1}F(\bar{a},\chi^{*})$$

and

3.8 $$\delta =R\left\{\frac{1}{\bar{a}}F(\bar{a},\chi^{*})+\frac{{\bar{a}}^{2}}{2}\right\}.$$

Here, *F*_1_ and χ* are fitting constants, $\bar{a}$ is a parameter which takes positive values, and $\bar{L}$ is a model parameter whose value (ratio of the brush thickness *L* to the effective radius *R*) is fixed from geometrical considerations.

We recall that equation (3.5) follows from equation (3.3), when it is solved for $\bar{F}$ as a function of $\bar{a}$. Now, let the numerical solution of equation (3.3) with respect to $\bar{a}$ be denoted as

3.9 $$\bar{a}=A\left(\frac{F}{F_{1}},\chi^{*}\right).$$

By adopting the usual notation $F^{-1}$ for the inverse function of the function F, we note that $A=F^{-1}$. In this way, equation (3.7) can be inverted to evaluate the value of the parameter $\bar{a}$ for a given value of the contact force $\bar{F}$. It is to emphasize that the model implementation is straightforward as it requires only numerically solving algebraic equations (for evaluating the function F and its inverse $F^{-1}$) in addition to nonlinear regression by least-squares method, which is routinely used in data fitting.

Hence, the substitution of equation (3.9) into equation (3.8) yields the relative indenter displacement *δ*/*R* as a function of the relative contact force *F*/*F*_1_, that is

3.10 $$\bar{\delta }=\frac{\bar{F}}{A(\bar{F},\chi^{*})}+\frac{1}{2}{\left[A(\bar{F},\chi^{*})\right]}^{2}.$$

After performing the fitting by minimizing the discrepancy between model prediction and data for *δ*/*R* using equation (3.10), the reduced modulus (in view of (3.1)_3_) can be evaluated as

3.11 $$E^{*}=\frac{3F_{1}}{8R^{2}},$$

whereas the other fitting constant χ*, which is defined by equation (3.2), yields the dimensional deformation parameter of the pericellular brush layer

3.12 $$p_{1}=\frac{4E^{*}}{\pi \chi^{*}},$$

where E* is already given by formula (3.11).

Figure 6 shows the processed *F*–*δ* curve (dotted line) along with the two fits according to the present model (curve 1), using equations (3.7) and (3.8), and the Hertz model (curve 2). Also, the post-prediction of the so-called steric model (curve 3) and its exponential approximation (curve 4) is shown based on equations (2.2) and (2.19).

In the dimensionless variables (3.1), the Hertzian equation (1.1) takes the form $\bar{F}=(1/2){\bar{\delta }}^{3/2}$. Since the Hertzian model becomes applicable in advanced stages of indentation, curve 2 in figure 6 was obtained by using the fitting formula $F=(F_{2}/2){\left(\bar{\delta }-{\bar{\delta }}_{2}\right)}^{3/2}$, where *F*_2_ is a dimensional scaling parameter, and ${\bar{\delta }}_{2}$ is a dimensionless offset parameter that accounts for the accumulated deformation of the pericellular brush. The corresponding prediction for the reduced elastic modulus is given by the formula E* = 3*F*_2_/(8*R*^2^), which is analogous to equation (3.11).

Finally, curves 3 and 4 in figure 6 were obtained based on Derjaguin’s approximations (2.19) and (2.20), applied for the AdG model (2.1) and (2.2) with the model parameters *p*_1_ and *L* evaluated by equations (3.12).

Based on the indentation brush/cell model (3.10), the following fitting results have been obtained for the assumed geometrical parameters *R* = 5 μm and *L* = 2.36 μm (average value for the brush thickness taken from [14]): *E*_1_ = 401.58 Pa, *p*_1_ = 15 Pa, and χ* = 45.4. At the same time, the Hertzian model-based fitting yields *E*_2_ = 586.5 Pa. Though we considered only one example based on a single data curve, the results would be qualitatively similar if we apply the model for the analysis of a number of other AFM indentation force–displacement curves for the same type of cells. In other words, the evaluated parameters E*, *p*_1_ and χ* should be regarded as indicative (approximate), as they were obtained without proper statistics.

Here and in what follows we shall refer to the ability of the constructed model to fit (more or less accurately) a set of data with a minimum number of adjustable parameters as its *robustness*. The robustness of the constructed model is demonstrated by fitting a set of experimental data for the whole range, including the brush-like behaviour, (figure 6) with the minimum of two adjustable (free) parameters (one for the brush layer and another one for the cell body). As it is seen from figure 6, the two limit models (the Hertzian model and the AdG brush model) provide accurate fitting only for certain ranges of the indenter displacement, which are not overlapping. In order to increase the quality of fit of the present model, it has the potential not only to increase the number of fitting parameters (e.g. exponents *ν*_1_ and *ν*_2_ of the constitutive function $f\;(\lambda )=\lambda^{-\nu_{1}}-\lambda^{\nu_{2}}$), but also to account for the bottom effect (by incorporating the characteristic cell thickness *H*). Namely, the latter effect seems to be responsible for the deviations at larger indentation depth, as the experimental force–displacement curve shows a more rapid increase with indentation.

**Figure 7.**
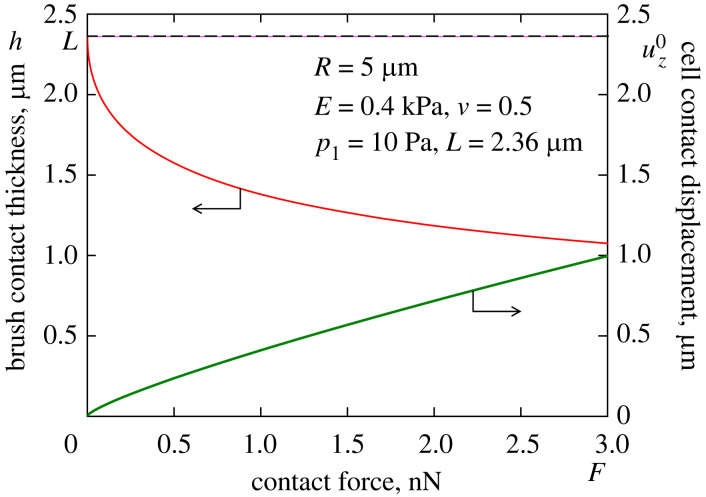
Brush contact thickness (left scale; upper red solid line) and cell contact displacement (right scale; lower green solid line) as functions of contact force for the indentation brush/cell model.

Figure 7 shows the variation of the contact parameters (2.27) and (2.28) for the evaluated values of the parameters of the brush/cell system. It is clearly seen from this figure that the pericellular coat (modelled in the framework of the AdG theory) is not completely squeezed out at the advanced stage of indentation.

Observe that the value of *E*_1_ is obtained to be somewhat lower than *E*_2_. The explanation for this phenomenon lies in the fact that the radius of curvature of the deformed brush/cell interface beneath the tip of the AFM probe is equal to *R* + *h*. If we take the latter value with *h* = 0.21 *L* (figure 7) that approximately corresponds to the advanced stage of indentation, then the corrected value will be *E*_2_ = 400.59 Pa, which is very close to the above provided value for *E*_1_.

## 4. Discussion and conclusion

First of all, let us discuss in more detail possible generalizations of the developed model. One of the ways to extend the range of applicability of the model is to adopt a more general theory of elasticity for the cell body. In particular, the elastic cell material can be assumed to be transversely isotropic. Provided the plane of elastic symmetry is parallel to the cell surface, equations (2.12), (2.24) and (2.25) will still apply, although the reduced elastic modulus E* should be modified accordingly (e.g. [13,34]).

Following [15], we can account for the effect of the cell prestress by modifying the elastic constant E*. We refer to papers [35,36] for more details. However, a more elaborated approach [27,37] is needed to take into consideration the effect of substrate on which a cell adheres. Following [38,39], a second-order asymptotic model can be worked out to account for a finite thickness of the cell body beneath the point of indentation.

It should be also noted that viscoelasticity is an intrinsic feature of the cell’s mechanical response to AFM indentation [40]. Following [13,41], the viscoelasticity of the cell body can be considered as well.

A generalization of the model for the case of conical [42] or monomial [43] indenters can be produced in a straightforward way, following the mathematical modelling approach outlined above. However, a special study is needed to account simultaneously for both the indenter shape effect [44] and the effect of spherical cell finite geometry [45].

Further, when regarding the role of the pericellular brush model (2.1) in the governing integral equation (2.12), it should be emphasized that equation (2.1) includes only two dimensional parameters *p*_1_ and *L*, while the constitutive function *f*(*λ*) is dimensionless. It goes without saying that experimental studies are required to determine a mechanobiologically motivated constitutive model for pericellular coat in compression.

We would like to underline that equation (2.11) tentatively employs the hypothesis of planar compression of the brush layer, which is strictly applicable for relatively thin brushes, i.e. for *L* ≪ *R*. It is pertinent to note here also that the Hertzian theory of contact employs a number of simplifying assumptions which are not always valid in practice. For example, the Hertzian force–displacement relation (1.1) assumes that *δ* ≪ *R* (small strains) and *a* ≪ *R* (paraboloidal approximation for the spherical indenter geometry). The latter restriction can be relaxed by adopting the geometrically exact equation for a spherical indenter, following the method developed in [28,46].

In addition, a remark should be made concerning previous developments in contact problems with interface effects. When the first (nonlinear) term on the left-hand side of equation (2.12) is linearized in the limit of relatively small pressures (when *p*(*r*) ≪ *p*_1_), equation (2.12) reduces to a linear Fredholm type integral equation of the second kind. Such a linear model describes the effect of a thin Winkler-type coating and was previously considered in [47]. If the nonlinear term in equation (2.12) is replaced with a power-law nonlinearity of the form $C{\left[p(r)\right]}^{\nu }$, the resulting nonlinear integral equation was studied using analytical methods in [48,49].

Finally, we note that any effects of adhesion (at the indenter/brush interface) and time-dependent deformation of both cell body [50] and brush layer [51] are neglected (indentation models for thin viscoelastic and biphasic layers have been developed in [52–54]), and thus, the modelling framework is applicable for quasi-static indentation only.

To conclude, the analytical model of the frictionless, non-adhesive, quasi-static AFM-based indentation of a living cell covered with a pericellular brush is shown to be robust in fitting experimental data and reveals a strong potential for further refinement.

## Data Availability

The datasets used and/or analysed during the current study are available from the corresponding author on reasonable request. The data are provided in electronic supplementary material [55].
